# Full-field hygro-expansion characterization of single softwood and hardwood pulp fibers

**DOI:** 10.1515/npprj-2020-0071

**Published:** 2020-12-12

**Authors:** N. H. Vonk, M. G. D. Geers, J. P. M. Hoefnagels

**Affiliations:** Department of Mechanical Engineering, 534526 Eindhoven University of Technology, Eindhoven, The Netherlands

**Keywords:** global digital height correlation, hygro-expansion, paper fiber, pulp, strain field

## Abstract

The dimensional stability of paper products is a well-known problem, affecting multiple engineering applications. The macroscopic response of paper to moisture variations is governed by complex mechanisms originating in the material at all length-scales down to the fiber-level. Therefore, a recently-developed method, based on Global Digital Height Correlation of surface topographies is here exploited to measure the full-field hygro-expansion of single fibers, i. e. a surface strain tensor map over the full field of view is obtained as function of time. From the strain field, the longitudinal and transverse hygro-expansion and principle strains can be calculated. Long- and intermediate-duration dynamic tests are conducted on softwood and hardwood fibers. A large spread in the softwood fiber’s transverse and longitudinal hygro-expansion coefficient ratio was found, while hardwood fibers behave more consistently. Computing the principle strain ratios reduces this spread, as it takes into account the variations of the deformation direction, which is directly affected by the micro-fibril angle (MFA). Furthermore, long-duration tests allow identification of the half-times at which the fibers equilibrate. Finally, the determined major strain angles for all fibers are consistent with the MFA ranges reported in the literature.

## Introduction

Softwood and hardwood pulp fibers are the main constituent in a wide range of paper-based products used for packaging, printing and converting industries. Their extreme sensitivity to moisture content variations is one of the major concerns for most applications. As generally known, an increase in moisture content greatly affects the mechanical and geometrical properties of the material at different length scales (Uesaka et al. 1992, Salmén 1993, Uesaka 2002, Wahlström 2009, Ganser et al. 2014, Linvill and Östlund 2014). During printing applications, a moisture content gradient throughout the thickness of the paper sheet causes undesired out-of-plane deformations, generally manifested as cockling, waviness or curling, strongly reducing the quality of the printed sheet. These unwanted out-of-plane deformations are governed by complex mechanism that originate in the fibrous micro-structure down to the single fiber level.

The hygro-expansion of single fibers or fibrils has been extensively studied by Tydeman et al. (1965), Meylan (1972), Nanko and Wu (1995), Weise and Paulapuro (1995) and Lee et al. (2010). Typical experiments consist of placing a sheet of paper, wet webs of pulp fibers or individual pulp fibers inside a climate chamber combined with an observation technique, e. g. X-ray projection imaging, atomic force microscopy, confocal scanning laser microscopy (CSLM) or digital light microscopy, allowing the measurement of moisture content induced dimensional changes. Salt solutions are often used to stepwise change the relative humidity (RH) in the specimen environment. Tydeman et al. (1965) compared micro-radiography images of wet webs to measure the global transverse shrinkage; Meylan (1972) used single fiber end-to-end tracking to measure the global longitudinal shrinkage as a function of the micro-fibril angle (MFA); Nanko and Wu (1995) used fiber-feature tracking on wet webs to measure the local average longitudinal strain during shrinkage; Weise and Paulapuro (1995) fitted ellipsoids to the CSLM images of the cross-section of paper fibers inside a paper sheet to measure the transverse shrinkage and Lee et al. (2010) used feature tracking to measure the local average longitudinal and transverse strain during shrinkage and swelling of pulp micro-fibrils. None of the previously mentioned works, besides the work by Lee et al. (2010), allow to determination of both the longitudinal and transverse shrinkage of the specimen. Yet, the experiments of Lee et al. (2010) were conducted on micro-fibrils and the achieved precision in both the longitudinal and transverse strain was rather low: $1\cdot 10^{-2}$ (where this number is obtained by fitting the data and determining the standard deviation). The specifics of the above-mentioned works concerning the longitudinal and transverse hygro-expansion magnitudes, pre-conditioning, testing range and specimen type can be found in Table 1. Combining the work of by Tydeman et al. (1965), Meylan (1972), Nanko and Wu (1995), Weise and Paulapuro (1995) leads to the general conclusion that single pulp fibers tend to swell 20–30 times more in transverse direction compared to the longitudinal direction (Wahlström 2009, Berglund 2012), but interestingly, none of the above mentioned works provide direct experimental evidence for this ratio of 20–30.

**Table 1:** Specifications of single fiber hygro-expansion experiments available in the current literature. MC: moisture content, RH: relative humidity, SW: softwood, HW: hardwood, FD: free drying, RD: restrained drying, BKP: bleached kraft pulp, *σ*: standard deviation.

Method	Longitudinal strain magnitude [-]	Transverse strain magnitude [-]	Pre-conditioning	Test range	Specimen type
Fiber-width tracking with micro-radiography (Tydeman et al. 1965)	not measured	0.18 (σ=0.05)	diluted fiber suspension	MC loss: 57 %	unbeaten SW pulp fibers
Fiber end tracking with universal length measuring machine (Meylan 1972)	0.002	not measured	2 days at each RH step	MC: 30 % to 0 %	unbeaten, unbleached SW fibers
Feature tracking with confocal laser scanning microscopy (Nanko and Wu 1995)	FD: 0.006 (σ=0.025)* RD: −0.012 (σ=0.020)*	not measured	Pressed wet webs	MC: 60 % to RH: 60 %	RD and FD handsheets of beaten, SW or HW BKP fibers
Ellipsoid fitting of fiber diameter using confocal scanning microscopy	not measured	0.40	not specified	MC: 33 % to 0 %	RD handsheets of BKP
Feature tracking with atomic force microscopy (Lee et al. 2010)	0.102 (σ=0.003)*	−0.037 (σ=0.100)*	one day at each RH step	MC loss: 2.86 %	aggregate micro-fibrils from SW BKP
Full-field correlation of surface height profiles [this work]	SW: 0.002 (σ=0.002) HW: 0.002 (σ=0.001)	SW: 0.056 (σ=0.007) HW: 0.047 (σ=0.005)	8 h at RH = 30 %	RH: 30 % to 90 %	unbeaten, SW and HW BKP fibers

^*^Mean and standard deviation are computed from the available data.

Full-field hygro-expansion data of single softwood and hardwood pulp fibers is called for, enabling the direct determination of both longitudinal and transverse hygro-expansion coefficients during absorption and desorption cycles. Moreover, this data enriched the parameter identification and provides in-depth insight in hygroscopic behavior. Full-field hygroscopic measurements are experimentally challenging, due to the fiber’s relatively low longitudinal hygro-expansion (maximally ∼2 % from dry to completely saturated (Meylan 1972)) combined with the fiber’s large kinematic freedom and large release of dried-in strain during wetting and drying, causing large displacement modes to occur, i. e. fiber rotation, bending and translation. Therefore, Vonk et al. (2020) have developed a experimental methodology, based on Global Digital Height Correlation (GDHC) of surface topographies which allows the determination of the time-resolved full-field hygro-expansion of single fibers, i. e. a map of the surface strain tensor over the full field of view as a function of time or relative humidity. The method’s robustness and high precision of $1.2\cdot 10^{-4}$ in longitudinal and $7.0\cdot 10^{-4}$ in transverse hygro-expansion was demonstrated for different natural and synthetic fiber types, each with different hygroscopic properties and measurement challenges.

Meylan (1972) and Yamamoto et al. (2001) have shown that the magnitude of longitudinal and transverse hygro-expansion of natural fibers is strongly affected by the MFA of the secondary fiber layer. While full-field data is desired, the major to minor strain angle can be determined and should be in adequate agreement with the MFA. Niskanen et al. (1997) performed long-duration hygroscopic experiments on paperboards which allowed identification of the half-time at which the hygroscopic strain comes to an equilibrium while the relative humidity is kept constant; in this work this half-time is referred to as the strain relaxation half-time. These half-times give a good indication of the timescale of paperboard to reach equilibrium when subjected to a change in relative humidity. While Niskanen et al. (1997) only determined the strain relaxation half-times of paperboards, the above-mentioned novel method enables the half-time identification of single fibers.

In this paper, the above-described novel method is applied to a series of softwood and hardwood pulp fibers, allowing identification of important fiber parameters, i. e. longitudinal and transverse hygro-expansion coefficients during wetting and drying cycles, strain relaxation half-times of absorption and desorption curves at constant relative humidity values. The validity of the proposed method’s capability to determine the MFA is also assessed. Due to the full-field time-resolved nature of the obtained data, all of these parameters can be extracted from one experiment on a single fiber. These parameters are not only useful for experimental applications, but also of key importance for the modeling of paper, i. e. homogenized fiber network models (Bosco et al. 2015, 2017), 3D beam network models (Motamedian and Kulachenko 2019) and level set based XFEM fiber network models (Samantray et al. 2020). Hence, this paper addresses the question whether one can determine, from a single experiment, the full-field hygro-expansion (and parameter identification) of single softwood and hardwood pulp fibers during wetting and drying cycles, allowing direct identification of the absolute longitudinal and transverse hygro-expansion magnitudes.

In order to establish an answer to this research question, first the considered method is explained, including, fiber preparation, testing and data processing. Subsequently, a comprehensive data analysis including parameter identification is conducted and the validity of the measurements is discussed, followed by conclusions.

## Materials and methods

**Figure 1:**
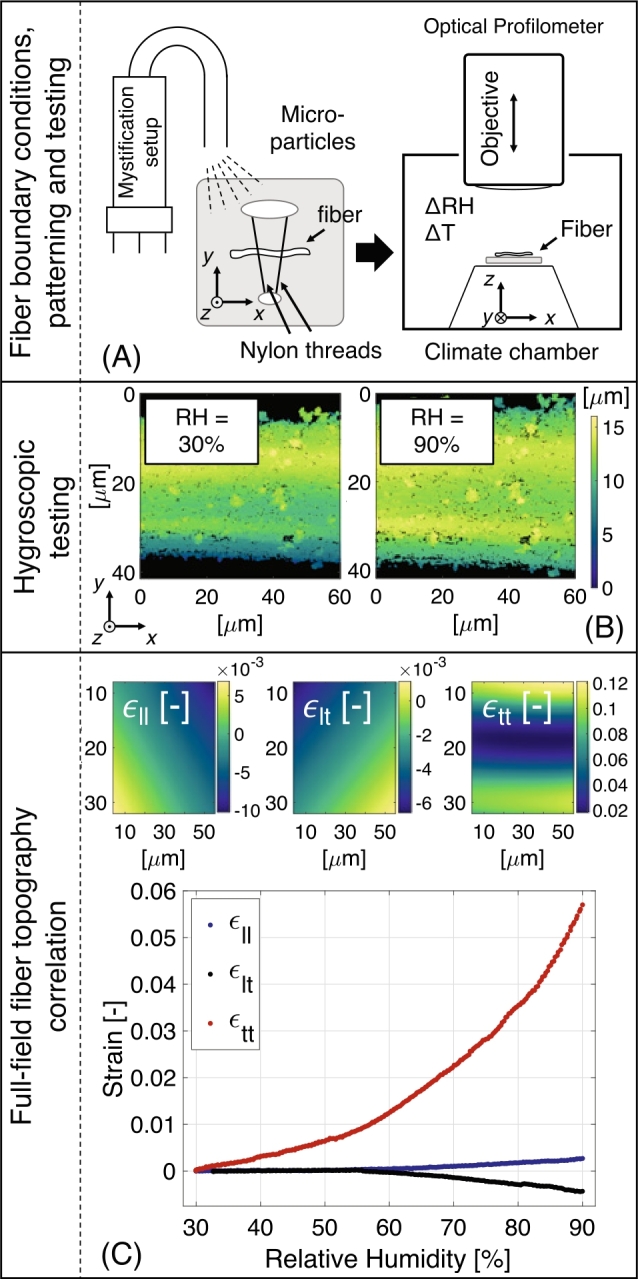
Schematic representation of the considered method with: (A) clamping of a single fiber which is decorated with micro-particles using a dedicated mystification setup, afterwards tested inside a climate box underneath an optical profilometer, (B) hygroscopic testing while taking consecutive fiber topographies to capture the fiber’s kinematics, (C) obtained topographies are processed using a Global Digital Height Correlation algorithm dedicated to fiber swelling, to obtain the average longitudinal ($\epsilon_{ll}$) and tangential strain ($\epsilon_{tt}$) of a single fiber which are plotted here.

The materials considered during the experiments are unbeaten fully bleached chemical softwood and hardwood pulp fibers (Kappa number 2) kindly provided by *Mondi Group*. The softwood pulp is a mixture of Spruce and Pine kraft pulp with an average length and width of, respectively, ∼2000µm and ∼30µm. The hardwood pulp is a eucalyptus kraft pulp with an average length and width of, respectively, ∼900µm and ∼20µm. The pulp is stored at a 50 % RH and 23 °C before testing. The fibers were extracted by delaminating the pulp bale following the procedure of Hirn and Bauer (2006) and pulling out the fibers that are naturally sticking out without touching or loading the region of interest. A total of five softwood and five hardwood fibers are tested throughout this research. The considered method that enables measuring the full-field hygro-expansion of single fibers, as proposed by Vonk et al. (2020), can be divided into three steps, schematically shown in Figure 1. Figure 1 (A) shows a single fiber fixed by two nylon threads, providing complete kinematic freedom for the hygro-expansion of the fiber while only reducing the global fiber’s rigid body motion (fiber swelling, bending and rotation are allowed). Prior to inserting the fiber into the climate chamber, a pattern of micro-particles (500 nm and 1µm) is applied on the fiber using a dedicated mystification setup (Shafqat et al. submitted for publication). This setup generates a mist of micro-particles immersed in ethanol which, after evaporation of the ethanol, is blown onto the fiber, resulting in a high-quality (unclustered) pattern without influencing the fiber surface. This pattern enables tracking of the surface displacements by means of GDHC of the evolving surface topographies of the fiber (Neggers et al. 2012, 2014). An external humidifier (*Cellkraft P-series Humidifier*) is used to control and register the relative humidity and temperature inside the climate chamber underneath an optical height profiler (*Bruker NPFlex*). The relative humidity is subsequently changed inside the climate box (in this work varied between a relative humidity of 30 and 90 %), while taking consecutive fiber topographies to extract the fiber’s kinematics, as shown in Figure 1 (B). Here, the obtained topographies are correlated using a special GDHC algorithm that is dedicated to fiber swelling, i. e. it accurately correlates the three-dimensional surface displacement field restricting the kinematic description to rigid body motion, homogeneous hygroscopic swelling, fiber rotation and fiber bending (corresponding to the minimal degrees of freedom of 18 (Neggers et al. 2012, 2014)). The GDHC results in full-field fiber surface displacement data, which is subsequently converted to a full-field fiber surface strain field. From this field, the average longitudinal ($\epsilon_{ll}$) and transverse ($\epsilon_{tt}$) surface strain can be computed and plotted versus relative humidity as shown in Figure 1 (C). For a more detailed description on the working principles, derivation, applicability and performance of the employed method, the reader is referred to Vonk et al. (2020).

To investigate the timescale at which a pulp fiber reaches its hygroscopic equilibrium, long-duration experiments are conducted on two softwood and hardwood fibers, with a relative humidity cycle of 30–90–30–90–30 %, where each setpoint is kept constant for 12 hours and a ramp of ±30 %/h is used to reach the next setpoint. Jajcinovic et al. (2018) have shown that, for a change from 0 % RH to 80 % RH, 40 mg of pulp needs approximately 6 hours at constant relative humidity to reach equilibrium. Therefore, a dwell time of 12 hours is adopted since tests are performed at 90 % RH, which may take longer.

To investigate the dynamic response of the single fiber, intermediate-duration experiments are conducted on three softwood and hardwood fibers, with a relative humidity cycle of 30–90–30–90–30–90–30–90–30 %, where each setpoint is kept constant for 2 hours and a ramp of ±30 %/h is used to reach the setpoints. This allows investigation of the hygroscopic dynamics during multiple absorption and desorption stages.

Before testing all fibers are equilibrated for 8 hours by keeping the relative humidity constant at 30 %. A topography is captured every 30 seconds by means of Vertical Scanning Interferometry, resulting in 240 topographies per setpoint switch from 30 to 90 % RH or back, which is an adequate amount of data to properly identify and correlate the fiber’s kinematics (Vonk et al. 2020). The softwood fibers are captured with a magnification of 100× reducing the image to a field of view (FOV) of $60\times 80\;\text{µ}{\text{m}}^{2}$ (1040 × 1376 pixels), whereas the hardwood fibers, due to their smaller width, are tested with an extra 2× tube lens, resulting in a magnification of 200×, reducing the FOV to $30\times 40\;\text{µ}{\text{m}}^{2}$. Finally, all tests are performed at a temperature of 23 ± 0.1 °C.

## Results and discussion

**Figure 2:**
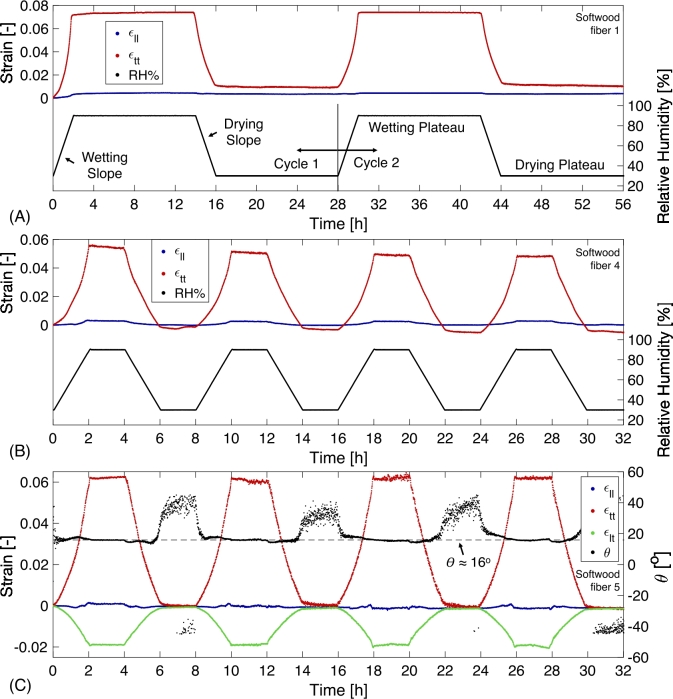
Hygroscopic strain response of three softwood fibers along with the measured relative humidity (right axis in (A) and (B)) with: (A) two long-duration cycles in which a clear relaxation trend is visible after the first wetting cycle at a constant relative humidity of 90 % and likewise during both drying cycles at a constant relative humidity of 30 %, (B) four intermediate duration cycles where the ongoing relaxation is visible during the constant relative humidity segments; and (C) the full-field hygroscopic response of four intermediate duration cycles, including the shear strain, subjected to the same relative humidity cycle as given in (B), together with the major strain angle (*θ*) plotted on the right axis, which seems to be constant around an angle of ∼16°, excluding small strain-difference regions, and may correspond to the MFA. (A) Indicates the stage denotations as used in the text.

The dynamic hygroscopic response of the long- and intermediate-duration tests of three softwood fibers is given in Figure 2 (A–C). As expected, the transverse hygro-expansion ($\epsilon_{tt}$) is much larger than the longitudinal hygro-expansion ($\epsilon_{ll}$) for all tests, which will be analyzed in detail below. Figure 2 (A) shows the transverse strain relaxation during the first wetting cycle, at 90 % RH, which saturates after approximately 8 hours. This relaxation trend is also visible during both drying cycles at 30 % RH. During the second wetting cycle, a plateau is visible which indicates that the fiber reached an equilibrium in the wet state after the first cycle. This behavior is also observed in the second long-duration test on another softwood fiber (not shown).

**Figure 3:**
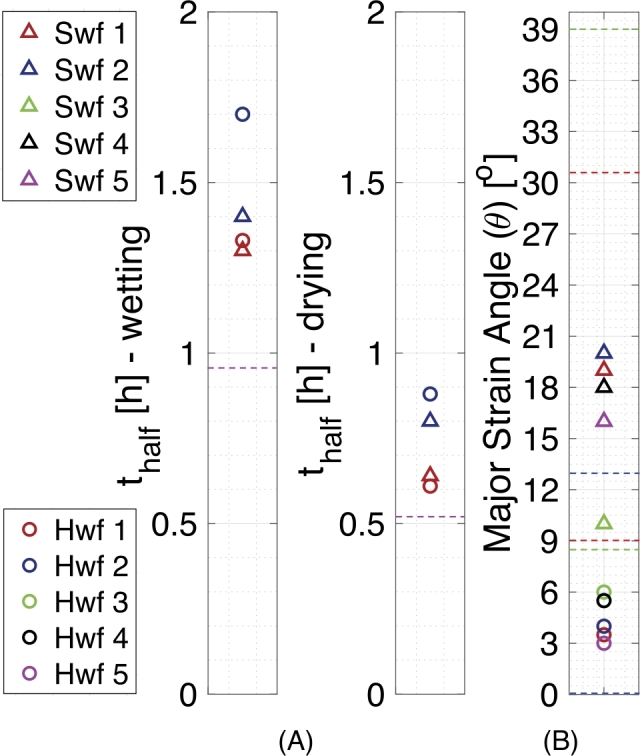
Parameter identification of the tested softwood and hardwood fibers with: (A) the transverse strain relaxation half-time ($t_{half}$) indicating the timescales at which the fibers equilibrate at 90 % RH (wetting) and 30 % RH (drying) whereby the half-time for adsorption and desorption found by Niskanen et al. (1997) on paper board scale are given by the dashed purple lines, (B) the major strain angle (*θ*) with the MFA ranges of the tested fibers given by the dashed lines; green: Spruce, red: Pine, blue: Eucalyptus. Fibers 1 - 2 correspond to the long-duration tests and fibers 3 - 5 to the intermediate-duration tests. Swf: softwood fiber, Hwf: hardwood fiber.

Figure 2 (B) shows an ongoing decreasing strain relaxation trend in transverse direction during both the wetting and drying cycles at respectively 90 % and 30 % RH, which saturates at high humidity levels after the fourth cycle during the wetting stage, i. e. in the order of ∼8 hours. The decreasing strain relaxation trend, however, contrasts the minor increasing strain trend in Figure 2 (A), which could imply the release of an initial dried-in strain that is present in the fiber before testing, which was also investigated on paper sheet scale by Smith (1950), Salmén et al. (1987) and Larsson and Wågberg (2008). Each fiber reveals a different initial dried-in strain, therefore both upwards and downwards strain trends may be found. More specific, permanent shrinkage in transverse direction is plausible, while in the inter-fiber bonded area the fibers are usually hindered to shrink in transverse direction by the low longitudinal hygro-expansion of the bonded fiber, this built-up stress is subsequently released upon wetting. However, permanent swelling of the fiber in transverse direction is less plausible, while it implies a compressive stress built up in the fiber during drying. An idea which may cause this is that the tested fiber was bonded to a parallel fiber (or a fiber under a shallow angle) which shrunk more upon drying, resulting in an initial compressive stress. Another less plausible idea is that the fiber was under longitudinal tension during drying, which enabled transverse compression of the fiber (Poisson effect) that is relaxed during wetting. However, this would lead to a negative longitudinal strain which is not observed. The half-time ($t_{half}$) have been extracted from the long-duration experiments by means of exponential fitting the transverse hygro-expansion trend at the wetting or drying plateau and are given in Figure 3 (A), in which fibers 1–2 correspond to the fibers subjected to long-duration tests. Note that the methodology’s strain precision ($1.0\cdot 10^{-4}$ and $2.1\cdot 10^{-4}$ in longitudinal and transverse direction, determined by of computing the standard deviation during the wetting plateau) was significantly increased compared to the values of $1.2\cdot 10^{-4}$ and $7.0\cdot 10^{-4}$ reported by Vonk et al. (2020). This is mainly attributed to the improved pattern quality, resulting from the applied dedicated mystification setup proposed by Shafqat et al. (submitted for publication).

Figure 2 (C) shows the hygroscopic response of a softwood fiber, subjected to an intermediate-duration relative humidity cycle, including the average surface shear strain ($\epsilon_{lt}$), to emphasize the full-field nature of the obtained strain data. Spectral decomposition of the surface strain tensor allows determination of the major and minor strain as well as the angle of the major strain axis in to the global coordinate system (*θ*), which starts at zero 90° from the longitudinal global fiber axis. In Figure 2 (C), *θ* tends to be constant around an angle of ∼16° (excluding the regions (30 < RH < 50 %) where the differences between $\epsilon_{ll}$, $\epsilon_{lt}$ and $\epsilon_{tt}$ are small, resulting in a large error in *θ*). This angle is good agreement with the micro-fibril angle ranges reported in the literature for the tested softwood fiber mixture; Spruce (8–39°) and Pine (9–31°) (Barnett and Bonham 2004, Cown et al. 2004). This major strain angle is computed for all the tested fibers and reported in Figure 3 (B), in which fibers 3–5 correspond to the intermediate-duration tests.

**Figure 4:**
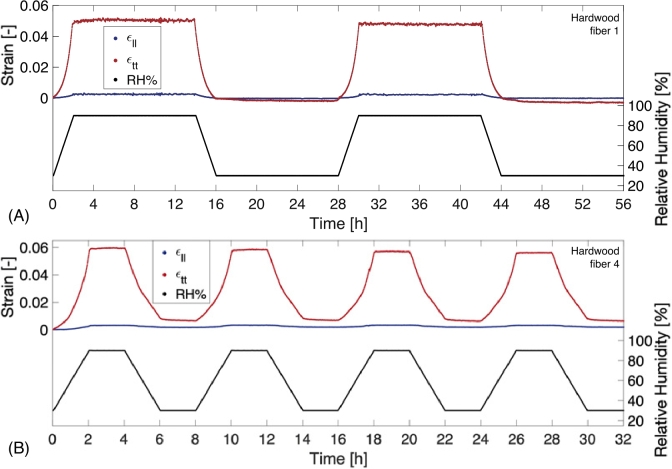
Hygroscopic strain response of two hardwood fibers with: (A) two long-duration cycles where a clearly visible relaxing trend after the first wetting cycle at a constant relative humidity of 90 % which equilibrates after a comparable timescale as the softwood fiber shown in Figure 2 and (B) four intermediate duration cycles where an overall relaxation is visible at a constant relative humidity of 90 and 30 %.

Figure 4 (A–B) shows the hygroscopic response of two hardwood fibers subjected to the long- and intermediate-duration tests. A transverse strain relaxation trend is visible in Figure 4 (A) at 90 % RH during the first wetting cycle and during both drying cycles at 30 % RH. The curve tends to equilibrate at a comparable timescale as for the softwood fibers. Figure 4 (B) shows the intermediate-duration hygroscopic response of a hardwood fiber, revealing an ongoing increasing transverse strain trend at 90 % and decreasing trend at 30 % RH. Both increasing and decreasing strain trends were found for different fibers, which implies that the above-described arguments causing this behavior (for softwood fibers) are also applicable for hardwood fibers. The half-times of the transverse strain relaxation trends are also reported in to Figure 3 (A). Additionally, the major strain angles of the tested hardwood fibers are computed and depicted in Figure 3 (B).

Figures 2 (A) and 4 (A) both show overall relaxation trends in transverse strain at 90 % and 30 % RH, of which the half-time ($t_{half}$) is extracted by means of exponential fitting, see Figure 3 (A). Note that the fiber’s moisture content and consequently the hygro-expansion show a bi-modal response following a step in relative humidity, i. e. the first major part of the response in moisture content occurs in less than a minute, however, the remaining response equilibrates over a time frame of hours (Jajcinovic et al. 2018). The considered method only captures the long-term behavior due to the rather long acquisition time of the microscope (30 seconds per scan). For softwood and hardwood fibers average half-times of, respectively, 1.35 and 1.52 hours are found during the wetting stages and, respectively, 0.72 and 0.75 hours for the drying stages. This is consistent with the work proposed by Niskanen et al. (1997) who found that softwood paperboards equilibrate faster during drying compared to wetting. Additionally, Niskanen et al. (1997) found half-times of approximately 1 and 0.5 hour at constant relative humidity levels of, respectively, 90 % and 30 % for different paperboards, which are marked in Figure 3 (A). However, these samples were subjected to an even steeper ramp in relative humidity (from 30 to 90 % and back) compared to the current research, therefore allowing the fibers to swell more rapidly and resulting in smaller half-times. The resulting values also comply with the equilibration moisture content half-time of around one hour found by Jajcinovic et al. (2018) on 40 mg of softwood pulp tested from 0 to 80 % RH. When comparing the half-times of softwood and hardwood fibers, no conclusive statements can be made.

As discussed before, the major strain angle is determined for all fibers and given in Figure 3 (B). For both softwood (Spruce and Pine) and hardwood (Eucalyptus) fibers this major strain angle complies with the ranges reported in the literature: Spruce (MFA: 8–39°) and Pine (MFA: 9–31°) and Eucalyptus (MFA 0–13°), as indicated by the dashed lines in Figure 3 (B) (Barnett and Bonham 2004, Cown et al. 2004, French et al. 2000, Donaldson 2008). This confirms that the method allows for proper determination of the major strain angle that is consistent with reported values of the MFA. Unfortunately, direct MFA validation experiments by means of other measurements techniques, i. e. X-ray Diffraction, small angle X-ray scattering, wide angle X-ray scattering and polarization microscopy were not available (Donaldson 2008).

**Figure 5:**
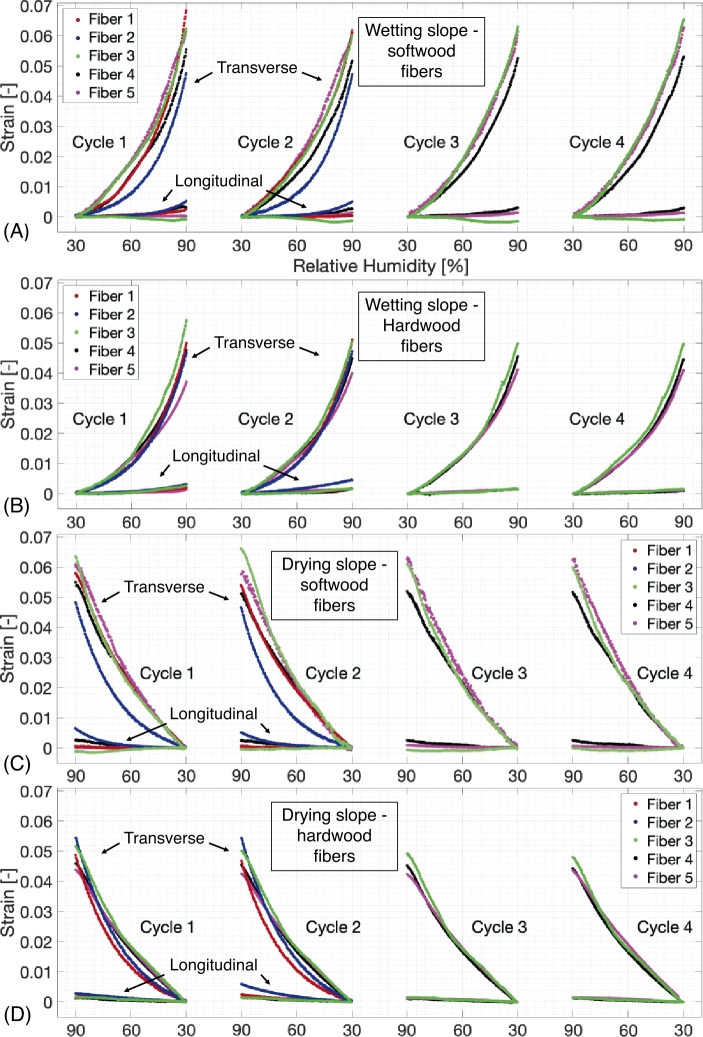
Shifted wetting and drying slopes of the tested softwood and hardwood fibers. A large variety in hygro-expansion is visible between all fibers. Repetitive hygroscopic behavior of the different fibers shows the consistency of the considered method. Softwood fibers show a much larger deviation in longitudinal hygro-expansion compared to hardwood fibers. The magnitude of hygro-expansion and the release of dried-in strain in both longitudinal and transverse direction are given in Appendix A.

Regarding the magnitude of the hygro-expansion; all wetting and drying cycles for both fiber types are extracted and plotted versus the relative humidity in Figure 5. To this end, for all wetting and drying curves are, respectively, the start and end points of the curve have been shifted to zero to enable an adequate comparison of the hygroscopic magnitude after multiple wetting or drying cycles. A significant variety in hygroscopic behavior is visible between different softwood and hardwood fibers during both wetting and drying, which was also observed on cellulose fibrils by Lee et al. (2010). For softwood fibers, the relative spread in longitudinal hygro-expansion during both wetting and drying is significantly larger than in transverse direction, which is not observed for the hardwood fibers. This can be explained by the wider range in MFA of softwood compared to hardwood fibers, respectively 8–39° Barnett and Bonham (2004), Cown et al. (2004) and 0–13° (French et al. 2000, Donaldson 2008), directly affecting the longitudinal hygro-expansion (Meylan 1972, Yamamoto et al. 2001). Variations in the sheet-scale hygro-expansion affected by the MFA of the fibers was also observed by Uesaka and Moss (1997). Furthermore, the considered method is highly consistent, i. e. no sign flipping of the longitudinal hygro-expansion is visible after multiple cycles for any of the fibers, e. g. softwood fiber 3 has a negative hygro-expansion in longitudinal direction, which remains negative for all subsequent wetting and drying cycles. This negative hygro-expansion may suggest that the swelling of the fiber is not solely dominated by the S2 layer, if the primary or the S1 layer are thick, they may induce the negativity that is found, this also affects the major principle strain angles given in Figure 3 (B). A negative longitudinal hygro-expansion was also observed on the viscose fibers tested in Vonk et al. (2020) and on cellulose fibrils in Lee et al. (2010). Therefore, the large fiber-to-fiber variability can be fully attributed to the difference in tested fibers and is properly captured through the high precision of the applied experimental methodology. While the average transverse strain increase (RH from 30 to 90 %) for all fibers is 0.052, the average longitudinal strain increase is 0.002. This is of comparable magnitude to the hygroscopic strains found on sheet scale by Uesaka et al. (1992); 0.003 and 0.005 for respectively restraint and freely dried handsheet subjected to a humidity cycle of 35 to 85 %. Additionally, as suggested by Uesaka (1994), the hygro-expansion in machine direction of anisotropic paper sheet is almost entirely dominated by the longitudinal hygro-expansion of the fibers, which again is supported by the average machine direction hygro-expansion of 0.0035 found by Niskanen et al. (1997) for a relative humidity cycle of 30 to 90 %. The magnitude of hygro-expansion in longitudinal and transverse direction for each wetting and drying slope shown in Figure 5 and the release of dried-in strain after each humidity cycle are given in, respectively, Tables 2 and 3 in Appendix A. Uesaka et al. (1992) have shown that during sheet level testing, an increase in hygro-expansion magnitude is visible after multiple humidity cycles. However, Table 2 shows that softwood and hardwood fibers 3–5, on average, reveal no clear increasing or decreasing trend. This suggest that other mechanisms, e. g. inter-fiber bond behavior, may play a significant role in the coupling of single fiber hygro-expansion to sheet-scale behavior.

**Figure 6:**
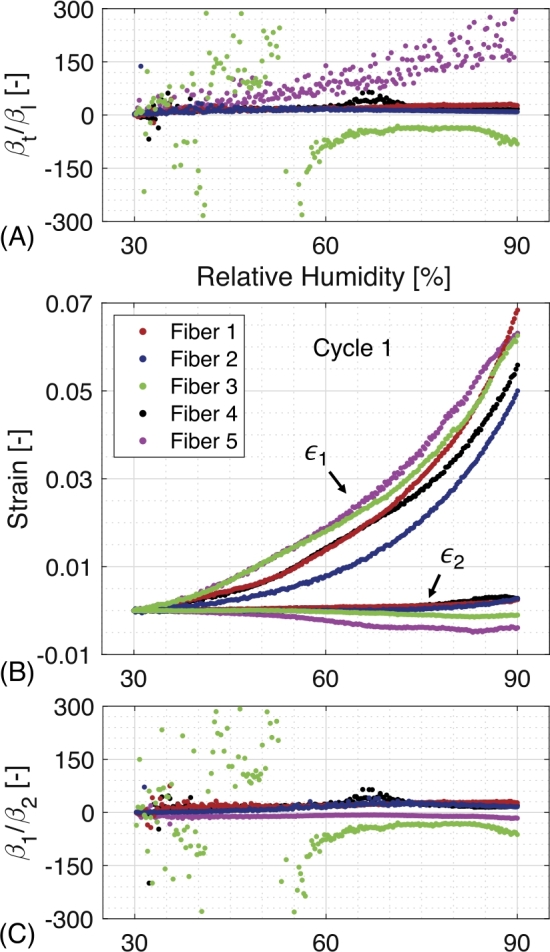
Hygro-expansion coefficient determination for the first wetting slope of the tested softwood fibers with: (A) the evolution of the ratio between the transverse and longitudinal hygro-expansion coefficient ($\beta_{t}/\beta_{l}$), (B) the major and minor principle strains and (C) the evolution of the ratio between the major and minor hygro-expansion coefficient ($\beta_{1}/\beta_{2}$) which shows significant less data noise and inter-fiber spread.

The hygroscopic strain curves shown in Figure 5 are subsequently converted into hygro-expansion coefficients. As the relation between the relative humidity and hygro-expansion is non-linear, it cannot be simply described using one coefficient for each direction. Therefore, the ratio between the transverse and longitudinal hygro-expansion coefficients ($\beta_{t}/\beta_{l}$) for the wetting and drying slopes of the tested softwood fibers are computed and $\beta_{t}/\beta_{l}$ for the first wetting slope is shown in Figure 6 (A). In the lower relative humidity region and subsequent lower strain region, large deviations are present, which tend to become constant after a relative humidity of 60 %. However, a significant spread exists between the different fibers. Computing the major and minor principle strain can reduce the spread visible in Figure 6 (A), whereby the principle strain direction will be affected by the MFA of the natural fiber (Meylan 1972, Yamamoto et al. 2001). The major and minor principle strain can be determined using a spectral decomposition of the strain tensor. Figure 6 (B) shows the principle strains of all softwood fibers’ first wetting cycle, which are subsequently converted into the ratio between the major and minor strain coefficient ($\beta_{1}/\beta_{2}$), given in Figure 6 (C). As expected, a significant decrease of, respectively, data noise in the lower relative humidity region and spread between the different fibers is visible when comparing Figure 6 (A) with (C).

**Figure 7:**
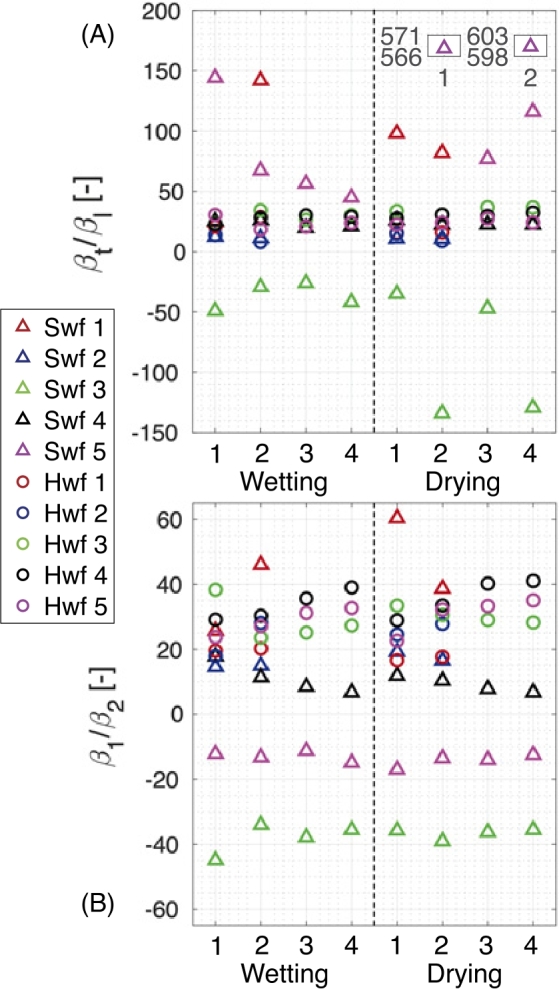
(A) The ratio between transverse and longitudinal hygro-expansion coefficient ($\beta_{t}/\beta_{l}$) with the larger ratios given in the small valued boxes. and (B) the ratio between the major and minor hygroscopic strain coefficient ($\beta_{1}/\beta_{2}$). Note that a negative hygro-expansion coefficient ratio results from a negative longitudinal hygro-expansion or minor strain. Fibers 1 - 2 correspond to the long-duration tests and fibers 3 - 5 to the intermediate-duration tests. Swf: softwood fiber, Hwf: hardwood fiber.

For both the principle and conventional hygro-expansion coefficient ratios, an average value is determined after a relative humidity of 60 % and are plotted in, respectively, Figure 7 (A) and (B). Note that a negative hygro-expansion ratio indicates a negative (small) longitudinal hygro-expansion coefficient. The hygro-expansion coefficient ratios for most of the fibers shown in Figure 7 (A) reside in a region of 10–50, which is comparable to the ratios reported in the literature (Wahlström 2009, Berglund 2012). However, some softwood fibers in Figure 7 (A) reveal significantly larger ratios and are highlighted in the small valued boxes. These larger ratios are also visible in Figure 6 (A) and are due to the small longitudinal hygroscopic strain which approaches zero, combined with the noise coming from the GDHC. Additionally, Figure 7 (A) shows that hardwood fibers behave more constant than softwood fibers, which is directly related to the smaller spread in longitudinal hygro-expansion visible in Figure 5. After determining the principle hygro-expansion coefficient ratios, Figure 7 (B) shows less spread between the fibers and also between the different wetting and drying cycles, clearly revealing the consistency of the obtained principle strain data see also Figure 6. Some fibers in Figure 7 (B) display a clear dried-in strain release in the first/second wetting cycle, e. g. for hardwood fiber 3 and softwood fiber 3, whereby the hygro-expansion coefficient ratio reaches a relatively constant value after the first wetting cycle. This dried-in strain release was also observed on eucalyptus and viscose fibers in Vonk et al. (2020). Finally, whereas Figure 7 (A) and (B) show the hygro-expansion ratios, Figure 5 can be used to assess the hygroscopic strain magnitude.

## Conclusion

Published literature to-date calls for new experimental data on the full-field hygro-expansion of pulp fibers, i. e. data which contains both longitudinal and transverse hygroscopic coefficients directly measured in a single experiment. A previously-developed method for the full-field identification of the hygroscopic properties of single fibers, of which the high precision and applicability has been demonstrated, is used in the current research. It involves minor clamping and micro-particle patterning of a single fiber, testing inside a climate chamber underneath an optical profiler and topography processing using a dedicated time-resolved Global Digital Height Correlation algorithm to obtain a complete surface strain field that adapts to the relative humidity. Long- and intermediate-duration cyclic experiments are conducted on both softwood and hardwood fibers to investigate the dynamic and saturation behavior.

The long-duration and intermediate-duration experiments reveal an overall relaxation trend at constant relative humidity levels of 90 % and 30 %. Upwards and downwards relaxation trends were found for both softwood and hardwood fibers, which suggests the release of an initial dried-in strain during wetting. The relative spread in the longitudinal hygro-expansion for softwood fibers is much larger than for hardwood fibers, which results from the wider range of micro-fibril angles (MFA) for softwood fibers, directly affecting the longitudinal hygro-expansion. All experiments allowed identification of the ratio between the transverse and longitudinal hygro-expansion coefficient. It was found that the hardwood fibers behave more consistently when subjected to multiple wetting and drying cycles, while the deviations for softwood fibers are significantly larger, which is again attributed to the larger range of MFA for softwood fibers. These deviations are, however, reduced when considering the ratio between the major and minor principle hygroscopic strain coefficient. For pulp fibers, this ratio is less affected by the MFA, resulting in a more constant value for softwood fibers, comparable to hardwood fibers. The long-duration tests allowed identification of the half-times during the constant relative humidity regimes. While there was no clear difference visible between the half-times of softwood and hardwood fibers, the resulting values are of comparable magnitude as reported in the literature on paperboards and fiber pulp. Due to the full-field nature of the data, a major strain angle is determined, which lies, for all tested fibers, within the MFA ranges reported in the literature. Hence, this indicated that the applied experimental methodology adequately identifies this important fiber parameter, obviously, more validation experiments are required to substantiate this.

In the proposed paper a series of softwood and hardwood fibers have been analyzed, using a versatile method that allows for single fiber parameter extraction, i. e. hygroscopic coefficients, half-times and major strain angles, which are essential for both quantitative experimental and modeling purposes.

## Appendix A

**Table 2:** The transverse ($\delta \epsilon_{tt}$) and longitudinal strain change ($\delta \epsilon_{ll}$) per wetting (30 to 90 % RH) or drying slope (90 to 30 % RH). Swf: softwood fiber, Hwf: hardwood fiber.

	wetting								drying							
		
cycle	(1)	(2)	(3)	(4)	(1)	(2)	(3)	(4)	(1)	(2)	(3)	(4)	(1)	(2)	(3)	(4)
		
	$\delta \epsilon_{tt}$ [-]	$\delta \epsilon_{tt}$ [-]	$\delta \epsilon_{tt}$ [-]	$\delta \epsilon_{tt}$ [-]	$\delta \epsilon_{ll}$ [-]	$\delta \epsilon_{ll}$ [-]	$\delta \epsilon_{ll}$ [-]	$\delta \epsilon_{ll}$ [-]	$\delta \epsilon_{tt}$ [-]	$\delta \epsilon_{tt}$ [-]	$\delta \epsilon_{tt}$ [-]	$\delta \epsilon_{tt}$ [-]	$\delta \epsilon_{ll}$ [-]	$\delta \epsilon_{ll}$ [-]	$\delta \epsilon_{ll}$ [-]	$\delta \epsilon_{ll}$ [-]
Swf 1	0.068	0.061	–	–	0.0053	0.0051	–	–	−0.049	−0.047	–	–	−0.0065	−0.0051	–	–
Swf 2	0.047	0.047	–	–	0.0026	0.0004	–	–	−0.058	−0.054	–	–	−0.0006	−0.0007	–	–
Swf 3	0.062	0.060	0.063	0.065	−0.0008	−0.0012	−0.0014	−0.0008	−0.064	−0.066	−0.061	−0.060	0.0012	0.0004	0.0007	0.0001
Swf 4	0.055	0.052	0.053	0.053	0.0032	0.0027	0.0030	0.0030	−0.055	−0.051	−0.052	−0.052	−0.0026	−0.0025	−0.0026	−0.0026
Swf 5	0.061	0.062	0.062	0.063	0.0003	0.0014	0.0013	0.0014	−0.061	−0.059	−0.063	−0.063	−0.0001	−0.0003	−0.0010	−0.0006
Hwf 1	0.047	0.047	–	–	0.0031	0.0047	–	–	−0.054	0.055	–	–	−0.0028	−0.0059	–	–
Hwf 2	0.050	0.051	–	–	0.0019	0.0017	–	–	−0.048	0.047	–	–	−0.0028	−0.0025	–	–
Hwf 3	0.057	0.051	0.050	0.050	0.0027	0.0016	0.0015	0.0015	−0.052	−0.051	−0.049	−0.048	−0.0015	−0.0014	−0.0015	−0.0013
Hwf 4	0.047	0.045	0.046	0.044	0.0020	0.0016	0.0015	0.0012	−0.046	−0.045	−0.045	−0.044	−0.0014	−0.0015	−0.0011	−0.0014
Hwf 5	0.037	0.040	0.041	0.041	0.0014	0.0017	0.0014	0.0012	−0.044	−0.043	−0.042	−0.044	−0.0017	−0.0015	−0.0012	−0.0014

**Table 3:** Release of dried-in strain after each humidity cycle in transverse ($\delta \epsilon_{tt}$) and longitudinal direction ($\delta \epsilon_{ll}$).

cycle	(1)	(2)	(3)	(4)	(1)	(2)	(3)	(4)
	
	$\delta \epsilon_{tt}$ [-]	$\delta \epsilon_{tt}$ [-]	$\delta \epsilon_{tt}$ [-]	$\delta \epsilon_{tt}$ [-]	$\delta \epsilon_{ll}$ [-]	$\delta \epsilon_{ll}$ [-]	$\delta \epsilon_{ll}$ [-]	$\delta \epsilon_{ll}$ [-]
Swf 1	0.0093	0.0016	–	–	0.0036	0.0001	–	–
Swf 2	−0.0024	−0.0009	–	–	−0.0004	−0.0001	–	–
Swf 3	−0.0074	−0.0071	0.0027	0.0026	0.0013	−0.0006	0.0007	−0.0009
Swf 4	−0.0015	−0.0019	−0.0021	−0.0001	−0.0001	−0.0001	0.0002	0.0000
Swf 5	0.0000	0.0002	−0.0003	0.0000	−0.0003	−0.0005	−0.0004	0.0000
Hwf 1	−0.0017	−0.0015	–	–	−0.0001	−0.0001	–	–
Hwf 2	−0.0235	−0.0051	–	–	−0.0098	0.0003	–	–
Hwf 3	0.0012	−0.0008	0.0000	−0.0006	0.0012	0.0001	−0.0001	−0.0003
Hwf 4	0.0064	0.0006	−0.0008	−0.0003	0.0017	0.0002	−0.0002	0.0000
Hwf 5	−0.0071	−0.0032	−0.0015	−0.0024	−0.0002	−0.0002	0.0002	−0.0002
